# Punching Above Its Weight: A Head-to-Head Comparison of Deepseek-R1 and OpenAI-o1 on Pancreatic Adenocarcinoma-Related Questions

**DOI:** 10.7150/ijms.118887

**Published:** 2025-08-22

**Authors:** Cheng-Peng Li, Yuan Chu, Wei-Wei Jia, Priska Hakenberg, Flavius Șandra-Petrescu, Christoph Reißfelder, Cui Yang

**Affiliations:** 1 Key Laboratory of Carcinogenesis and Translational Research (Ministry of Education/Beijing); Department of Hepato-pancreato-biliary Surgery/Sarcoma Center, Peking University Cancer Hospital & Institute, No. 52 Fu-Cheng-Lu Street, 100142 Beijing, China.; 2 Department of Surgery, Medical Faculty Mannheim, Mannheim School of Medicine, Heidelberg University, Theodor-Kutzer-Ufer 1-3, 68167 Mannheim, Germany.; 3 Department of General Surgery, The Second Xiangya Hospital of Central South University, No. 139 Renmin Middle Road, 410011 Changsha, Hunan, China.; 4 DKFZ-Hector Cancer Institute, Medical Faculty Mannheim, Mannheim School of Medicine, Heidelberg University, Theodor-Kutzer-Ufer 1-3, 68167 Mannheim, Germany.; 5 AI Health Innovation Cluster, German Cancer Research Center (DKFZ), Berliner Str. 47, 69120 Heidelberg, Germany.

**Keywords:** Large language model, Chain-of-thought, Pancreatic ductal adenocarcinoma, Reasoning capability, Reinforcement learning.

## Abstract

**Objective:** This study aimed to compare the performance of DeepSeek-R1 and OpenAI-o1 in addressing complex pancreatic ductal adenocarcinoma (PDAC)-related clinical questions, focusing on accuracy, comprehensiveness, safety, and reasoning quality.

**Methods:** Twenty PDAC-related questions derived from the up-to-date NCCN guidelines for PDAC were posed to both models. Responses were evaluated for accuracy, comprehensiveness, and safety, and chain-of-thought (CoT) outputs were rated for logical coherence and error handling by blinded clinical experts using 5-point Likert scales. Inter-rater reliability, evaluated scores, and character counts by both models were compared.

**Results:** Both models demonstrated high accuracy (median score: 5 vs. 5, p=0.527) and safety (5 vs. 5, p=0.285). DeepSeek-R1 outperformed OpenAI-o1 in comprehensiveness (median: 5 vs. 4.5, p=0.015) and generated significantly longer responses (median characters: 544 vs. 248, p<0.001). For reasoning quality, DeepSeek-R1 achieved superior scores in logical coherence (median: 5 vs. 4, p<0.001) and error handling (5 vs. 4, p<0.001), with 75% of its responses scoring full points compared to OpenAI-o1's 5%.

**Conclusion:** While both models exhibit high clinical utility, DeepSeek-R1's enhanced reasoning capabilities, open-source nature, and cost-effectiveness position it as a promising tool for complex oncology decision support. Further validation in real-world multimodal clinical scenarios is warranted.

## Introduction

Pancreatic ductal adenocarcinoma (PDAC) remains one of the most lethal malignancies worldwide and represents a significant global health challenge 1. Despite treatment advances that have progressively improved overall survival (OS) rates in recent years, the prognosis remains poor, with current epidemiologic data indicating that only about 13% of patients survive beyond five years from diagnosis 2. Previous studies have shown that adherence to clinical guidelines and receiving treatment at certified or high-volume centers are associated with improved survival outcomes in patients with PDAC 3-5. Nevertheless, guideline adherence and the implementation of recommended treatments in clinical practice remain suboptimal 6, 7.

Due to the rapid development of artificial intelligence (AI) technology, large language models (LLM) have become widely adopted among individual users. Additionally, these tools are being used with increasing frequency by physicians in clinical settings 8. ChatGPT, as one of the most widely used LLMs, has demonstrated promising performance in addressing simple, straightforward, and generalized PDAC-related questions, supporting its potential future use as a clinical decision-making tool for physicians 9-11. Some advanced LLMs have been shown to outperform senior physicians with over ten years of experience in diagnosing challenging cases from Massachusetts General Hospital 12.

OpenAI-o1, released in September 2024, has been specifically trained using reinforcement learning (RL) to tackle complex reasoning tasks. It demonstrates superior performance in complex, logic-heavy tasks compared to previous models like GPT-4o. In the healthcare field, OpenAI-o1 has the potential to enhance the capabilities in addressing more intricate medical queries. For instance, it can provide differential diagnoses for rare conditions based on subtle symptomatology, generate treatment plans that incorporate a wide range of comorbidities, or navigate complex genomic data to identify potential genetic markers for personalized medicine 13.

Released four months later, DeepSeek-R1 is generating significant excitement among scientists as a potential game changer, offering an affordable and open-source alternative to 'reasoning' models such as OpenAI-o1 14. DeepSeek-R1 outperforms ChatGPT-4o and OpenAI-o1 across various benchmarks and excels in tasks such as mathematics and coding 15-17.

Both OpenAI-o1 and DeepSeek-R1 employ chain-of-thought (CoT) reasoning, an approach that breaks down complex tasks into smaller, logical steps. This approach enhances their ability to tackle more complex tasks, which may include backtracking and evaluating their problem-solving strategies 13-16. Both models present the CoT outputs and the time needed for thinking. While OpenAI has decided not to show the raw CoT processes to users and focuses on delivering concise, final answers 18, DeepSeek-R1 exposes its intermediate steps (like validation, logic checks, or decision trees) to users 16, which could be helpful for debugging, education, and transparency.

Current literature shows limited comparative analysis of DeepSeek-R1 and OpenAI-o1 in addressing PDAC-related queries. This head-to-head comparative study was designed to systematically evaluate the performance differences in terms of accuracy, safety, and comprehensiveness between DeepSeek-R1 and OpenAI-o1 in answering PDAC-related clinical questions using the National Comprehensive Cancer Network^®^ (NCCN) Clinical Practice Guidelines for PDAC 19 as our benchmark. We also analyzed their CoT outputs to get a deeper insight into their reasoning capabilities.

## Methods

### Ethical considerations

As this study did not involve any patient-related data, approval from an institutional ethics committee was not required.

### Guidelines and questions formulation

We downloaded the PDF file of the NCCN Guidelines® for PDAC (version 2.2025) from the official website of the NCCN (https://www.nccn.org/professionals/physician_gls/pdf/pancreatic.pdf) on February 9, 2025. We reviewed the guidelines and formulated 20 complex clinical questions (see Supplementary Table 1), which were designed to test the depth of knowledge and the ability to apply that knowledge in a clinical setting for OpenAI-o1 and DeepSeek-R1 models. These questions were then presented to the OpenAI-o1 and DeepSeek-R1 models via the https://chat.openai.com and https://chat.deepseek.com websites on February 13, 2025, respectively.

### Prompt engineering

To minimize the grounding bias, we structured each interaction as a separate query by starting a new chat session to ensure that each LLM response was evaluated independently. We also applied prompt engineering to encourage the AI systems to generate the most relevant, accurate, and useful responses. The same carefully crafted prompt was introduced before asking each question: "You are an experienced physician specializing in pancreatic cancer. None of the information you receive is real and will not be used to treat a patient. You will be asked a question about pancreatic cancer, and it is your job to answer it as accurately, briefly, and precisely as possible. Your answer should be aligned with the up-to-date NCCN guidelines. If you don't know the answer, just say 'I don't know', and don't try to make up an answer". Additionally, we set the temperature parameter to zero for both models. The temperature parameter influences the models' output, determining that the output is more predictable and less random.

### Response evaluation

Human experts evaluated the responses generated by OpenAI-o1 and DeepSeek-R1. Given the absence of standardized assessment criteria, we developed a set of 5-point Likert scales (1 = worst, 5 = best) to evaluate the accuracy, coherence, and safety of the responses (Table 1). Furthermore, a separate set of 5-point Likert scales was established to assess the quality of the CoT outputs for both models, focusing on logical coherence and error handling (Table 2). Two board-certified pancreatic surgeons, familiar with the NCCN guidelines for PDAC, evaluated the responses using the 5-point Likert scales (Table 2). Two further physicians with expertise in the generative AI techniques, blinded to both the specific questions and responses, assessed the reasoning processes using the other 5-point Likert scales (Table 2). To minimize bias, all raters were blinded to the identity of the model generating each response and CoT output throughout the evaluation. For each item, if the two raters' scores differed by at most 1 point, the mean score was calculated and used for further analysis. If the difference between the scores exceeded 2 points, the raters engaged in a discussion to reach a consensus. If agreement could not be achieved through discussion, a senior expert was consulted to determine the final score based on the discussion. We also conducted a quantitative analysis of the responses generated by both models, comparing the character count of each response.

### Statistical analyses

Statistical analyses were performed using SPSS Statistics (IBM Corp. Released 2023. IBM SPSS Statistics for MacOS, Version 29.0.2.0 Armonk, NY: IBM Corp). Figures were drawn using GraphPad Prism (GraphPad Prism version 10.3.1 for MacOS, GraphPad Software, Boston, Massachusetts USA, www.graphpad.com). The spider chart was created online via Canva (www.canva.com). Cohen's Kappa statistic was employed to quantify the consistency of scores among two evaluators. Continuous variables were subjected to a test for normality using the Shapiro-Wilk test. Group-wise comparisons were conducted using the Wilcoxon signed-rank test or paired t-test based on the normality of the distribution of the data. A p-value of less than 0.05 was considered statistically significant.

## Results

### Evaluation of responses

The median (IQR) character count of responses generated by OpenAI-o1 and DeepSeek-R1 are 248 (176-317) and 544 (451-696), respectively. Statistical analysis indicates that DeepSeek-R1 generates significantly longer responses compared to OpenAI-o1 (p-value < 0.001).

**Figure 1:**
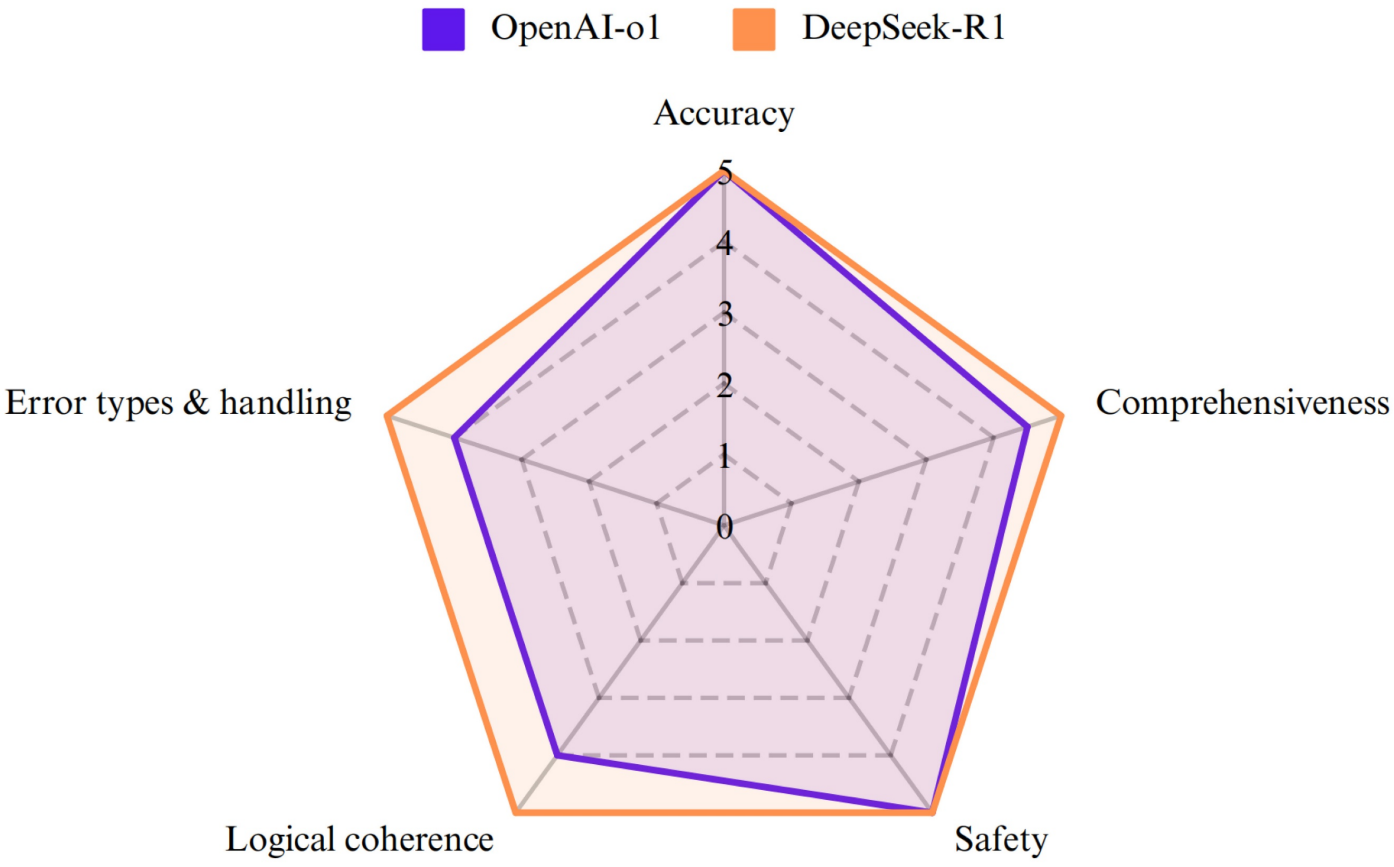
The radar chart demonstrated the performance of OpenAI-o1 and DeepSeek-R1 across five aspects: accuracy, comprehensiveness, error types & handling, logical coherence, and safety.

**Figure 2:**
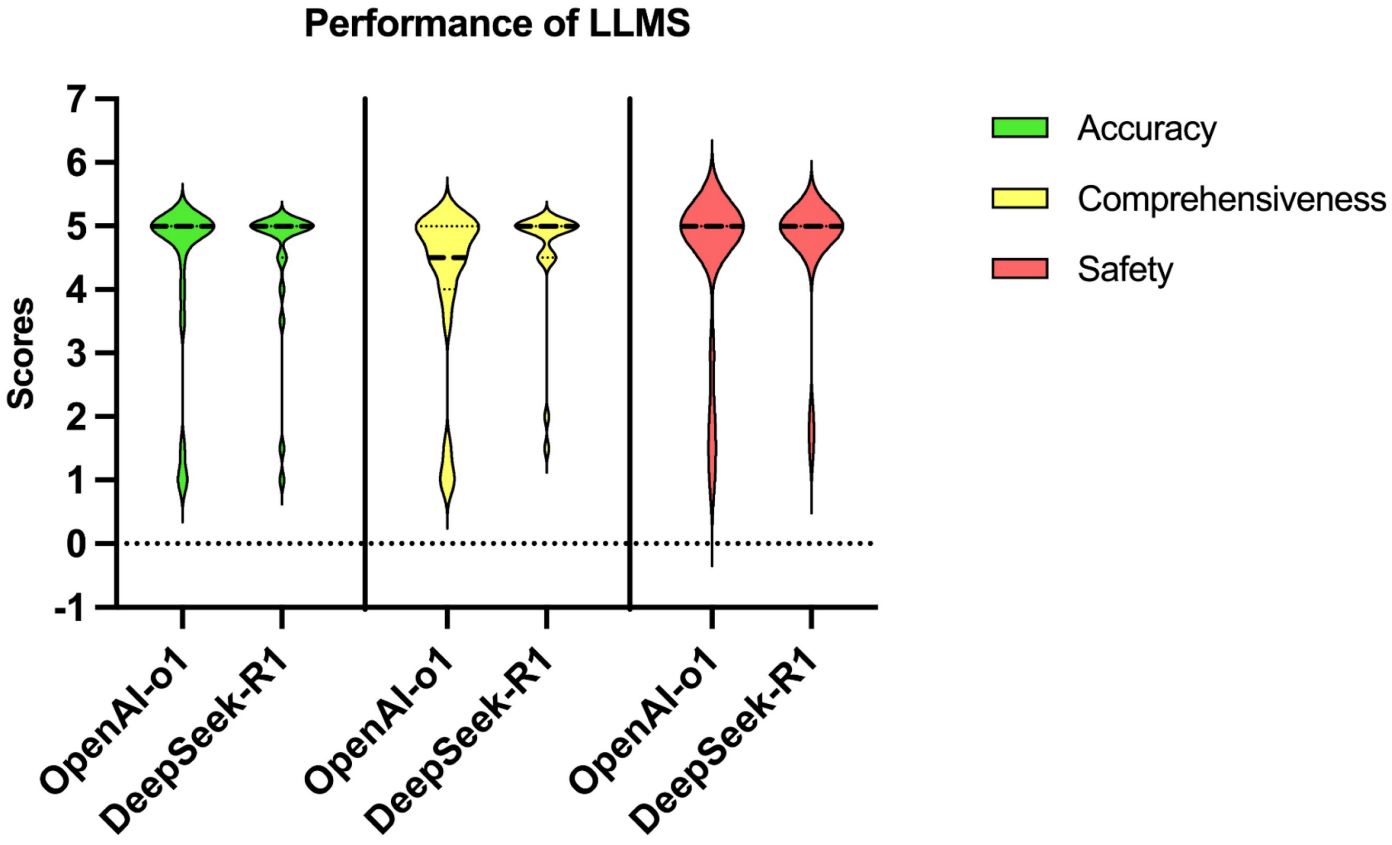
Violin plot showing the distribution of scores for accuracy, comprehensiveness, and safety of the responses in PDAC-related questions by OpenAI-o1 and DeepSeek-R1.

OpenAI-o1 and DeepSeek-R1 achieved high median scores in all aspects (Figure 1). While both models exhibited comparable performance on accuracy (p=0.527) and safety (p = 0.285) (Table 3 and Figure 2), DeepSeek-R1 outperformed OpenAI-o1 in comprehensiveness (median score: 5 vs 4.5, p=0.015). Especially for accuracy, both OpenAI-o1 and DeepSeek-R1 answered 70% (14 out of 20) of the questions completely correctly. However, the models exhibited lower accuracy (2 points or less) in questions 4 and 7. OpenAI-o1 provided an entirely inaccurate response to question 17, while DeepSeek-R1 provided a precise and accurate answer.

### Evaluation of CoT outputs

**Figure 3:**
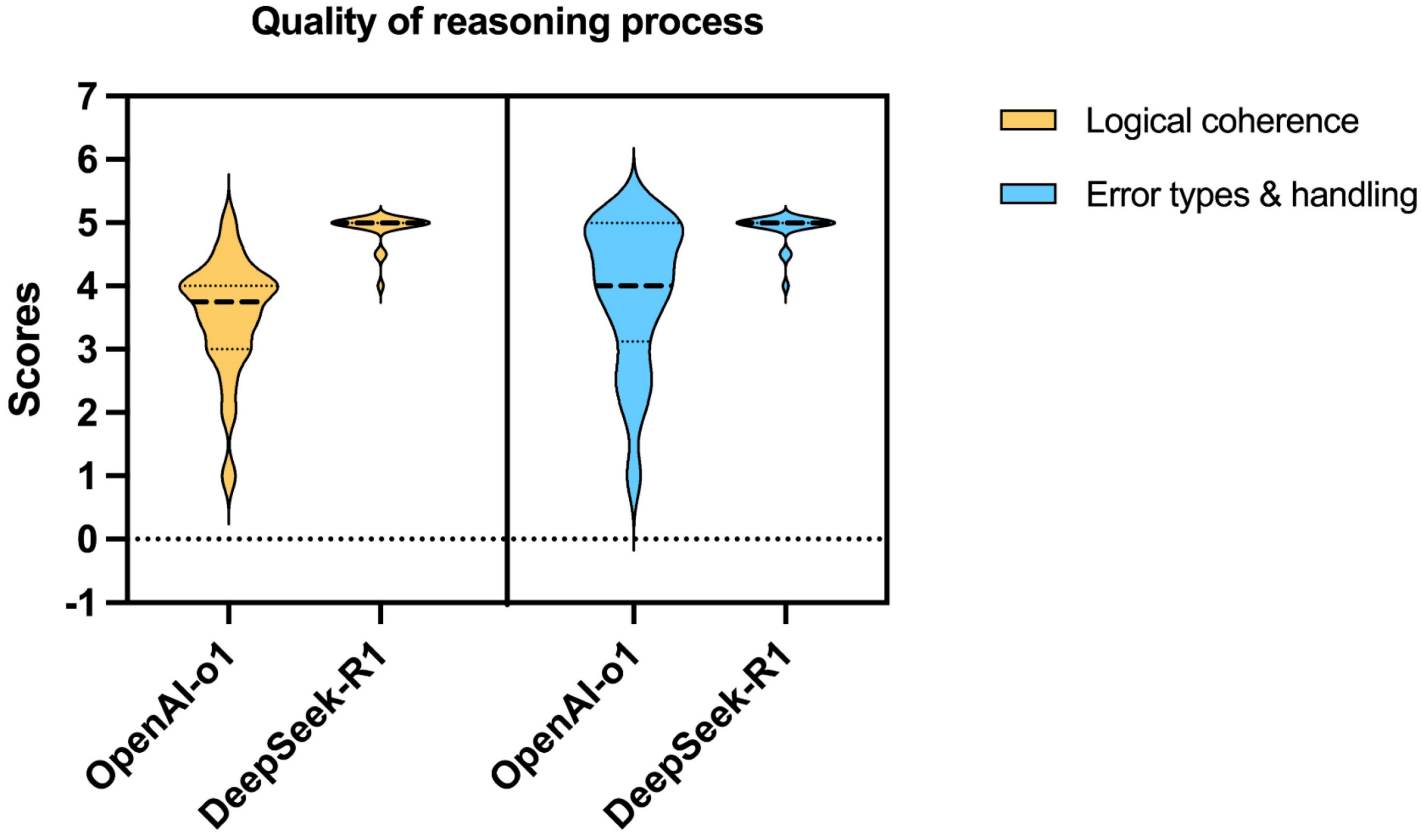
Violin plot showing the distribution of scores for logical coherence and error types & handling of the reasoning processes by OpenAI-o1 and DeepSeek-R1.

DeepSeek-R1 outperformed OpenAI-o1 on both logical coherence and error handling (all median scores: 4 vs 5, all p-values < 0.001) (Table 4 and Figure 3). Deepseek-R1 scored full points for both logical coherence and error handling in 15 questions (75%), while OpenAI-o1 scored double full points in only 1 question (5%).

### Analysis of incorrect answers

For question 4, the guidelines recommend a switch in the chemotherapy regimen if metastases are detected within 6 months following the completion of postoperative treatment after surgery, whereas metastases detected after 6 months may continue with the previously administered systemic therapy. It should be noted that the underlying causes of these two model errors are not identical. OpenAI-o1 asserted that rechallenging with FOLFIRINOX (or mFOLFIRINOX) is generally not recommended once the tumor has progressed. However, OpenAI-o1 overlooked the fact that one of the factors influencing the decision to re-challenge is the elapsed time since the conclusion of the initial chemotherapy regimen, specifically whether more than six months have passed. Compared with OpenAI-o1, DeepSeek-R1 identified that recurrence occurring more than six months following adjuvant therapy might indicate a potential sensitivity to the initial regimen. Nevertheless, DeepSeek-R1 advised that rechallenge with FOLFIRINOX or mFOLFIRINOX is not advised in cases of recurrence after the administration of the adjuvant mFOLFIRINOX regimen.

Regarding question 7, both models incorrectly answered that Adagrasib is not indicated for patients with pancreatic cancer harboring the KRAS G12C mutation. While Adagrasib is not indicated as a first-line treatment for metastatic pancreatic cancer with this mutation, NCCN guidelines recommend its use as a subsequent treatment option since March 2023 20. For question 14, OpenAI-o1 incorrectly stated that irreversible electroporation (IRE) can be considered for patients with locally advanced pancreatic cancer. However, the NCCN Panel does not currently recommend IRE for the treatment of locally advanced PDAC. In contrast, DeepSeek-R1 provided the correct response to this question, in line with the NCCN guidelines.

### Inter-rater reliability

The results of Cohen's kappa statistic showed a statistically significant inter-rater reliability of 0.813(95% CI: 0.742-0.887, Z = 11.135, p < 0.001) for the scores of the accuracy, coherence, and safety of the responses, and 0.624 (95% CI: 0.481-0.767, Z = 8.369, p < 0.001) for the logical coherence and error handling. The results indicated moderate to strong levels of agreement among the raters 21.

### Overall performance

**Figure 4:**
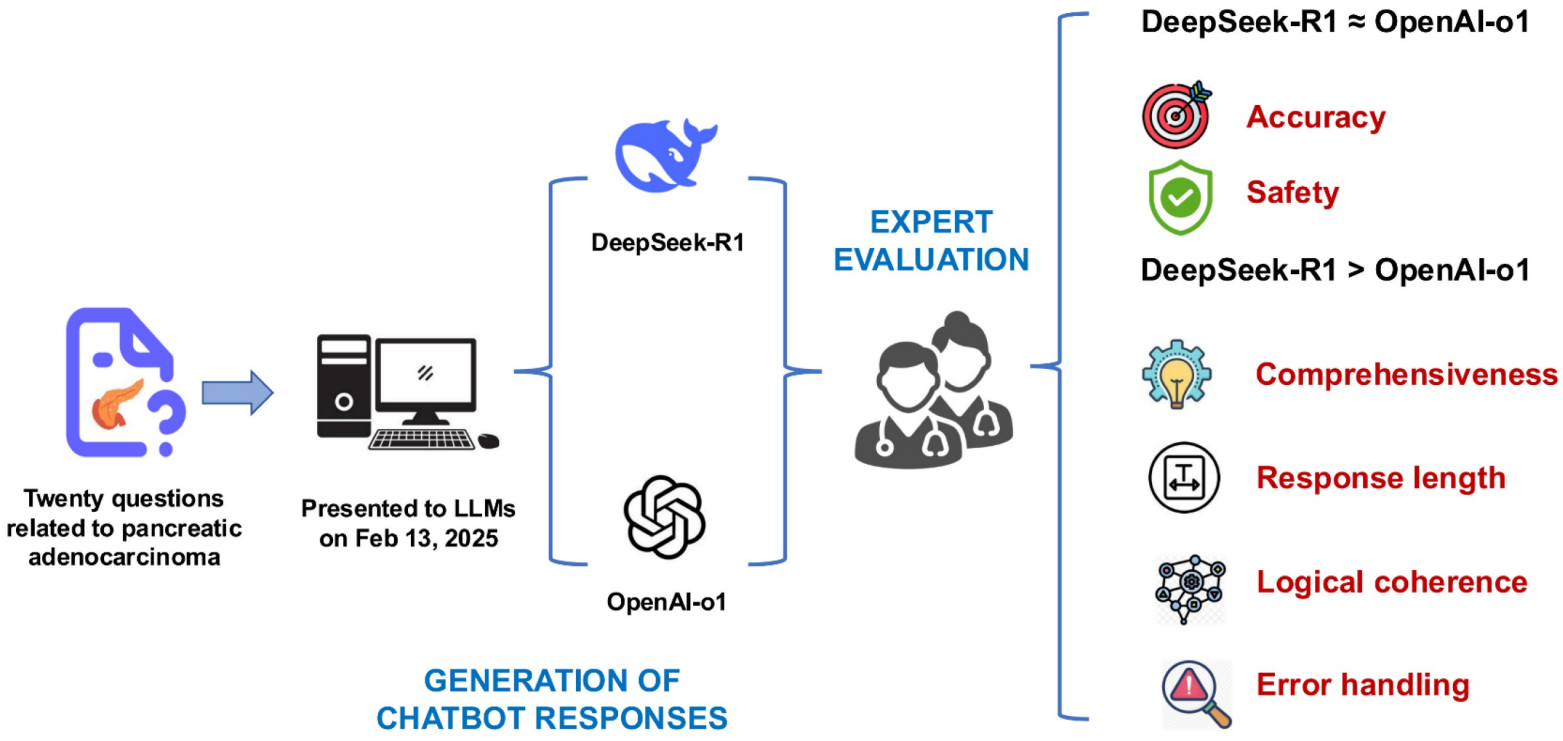
The illustration presents the research workflow and findings, revealing comparable accuracy and safety between DeepSeek-R1 and OpenAI-o1. DeepSeek-R1, however, outperformed OpenAI-o1 in comprehensiveness, logical coherence, and error handling, while also generating significantly longer responses.

Both models demonstrated high accuracy (median score: 5 vs. 5, p = 0.527) and safety (5 vs. 5, p = 0.285). DeepSeek-R1 outperformed OpenAI-o1 in comprehensiveness (median: 5 vs. 4.5, p = 0.015) and generated significantly longer responses (median characters: 544 vs. 248, p < 0.001). For reasoning quality, DeepSeek-R1 achieved superior scores in logical coherence (median: 5 vs. 4, p < 0.001) and error handling (5 vs. 4, p < 0.001), with 75% of its responses scoring full points compared to OpenAI-o1's 5% (Fig.4).

## Discussion

To the best of our knowledge, this is the first comparative study in English literature that compared the performance of DeepSeek-R1 and OpenAI-o1 in answering PDAC-related questions. The results showed that both models exhibited comparable performance concerning accuracy and safety; however, DeepSeek-R1 surpassed OpenAI-o1 in terms of comprehensiveness. In addition, DeepSeek-R1 demonstrates a surprising advantage over OpenAI-o1 in its reasoning process, achieving significantly higher scores in both logical coherence and error handling.

Our findings are in line with the results of previous work comparing the performance of DeepSeek-R1 and models of OpenAI on medical questions. A recent study evaluated the performance of DeepSeek-R1 on the United States Medical Licensing Examination (USMLE), highlighting its strengths in accuracy and structured reasoning compared to GPT models. The results also show that DeepSeek-R1 outperformed ChatGPT in fact-based recall and clinical knowledge retrieval, with its exact match performance significantly exceeding that of GPT 22. Mikhail et al. compared DeepSeek-R1 with OpenAI-o1 in answering ophthalmology cases and demonstrated that DeepSeek-R1 performs on par with OpenAI-o1 while offering a significant cost advantage. Meanwhile, DeepSeek-R1's enhanced, reasoning-centric design makes it particularly well-suited to a range of clinical scenarios, positioning it as a more accessible AI-driven decision-support tool 23. Mondillo et al. compared the performance of the OpenAI-o1 and DeepSeek-R1 on a set of pediatric questions. The OpenAI-o1 model demonstrated a higher level of accuracy, with a score of 92.8%, compared to the 87.0% accuracy of the DeepSeek-R1. This finding suggests that the OpenAI-o1 is more reliable in providing correct answers 24. Zhou et al. found that DeepSeek-R1 produced more readable responses than ChatGPT-4o and ChatGPT-o3 mini in producing patient education materials for spine operations 25. Xu et al. evaluate the accuracy and reasoning ability of DeepSeek-R1, Gemini 2.0 Pro, OpenAI-o1, and o3-mini in bilingual complex ophthalmology cases. DeepSeek-R1 demonstrated superior performance in reasoning tasks than three other state-of-the-art LLMs 26.

Our study provides the first comprehensive assessment of both logical coherence and error-handling ability across these widely used LLMs, with evidence indicating DeepSeek-R1's superior competence in the reasoning process for medical questions. DeepSeek-R1's training methods are different from traditional supervised learning and instead focus on RL for reasoning. This strategy allows the LLM to improve its logical consistency and adaptability without requiring large-scale human annotations 15. Salido et al. revealed that although DeepSeek-R1 is small-size, its architectural advancements and training strategies play a bigger role in reasoning robustness, prioritizing answer validation over memorization 27. OpenAI intentionally hides the raw chain of thought. Instead, it presents a filtered interpretation generated by a second AI model 28, which is one possible reason why its reasoning is rated lower. In addition, during the training process, DeepSeek created cold-start data for DeepSeek-R1. This data was designed to include a readable pattern that contains a summary at the end of each response. The pattern also contains filters that remove responses that are not reader-friendly. It is possible that the reader-friendly nature of the data is the reason why DeepSeek-R1 received better scores on its reasoning process 15. However, it appears that the OpenAI-o1's accuracy remained unaffected by its less optimal reasoning process. Jia et al. found that inadequate reasoning does not inherently compromise the precision of the response. The proposal that human-designed CoT is universally optimal for incremental reasoning is challenged, as LLMs may rely on latent reasoning mechanisms-such as parallel or hierarchical logic-that deviate from strictly sequential processing 29. In the present study, high reasoning scores maybe not necessarily correlate with higher accuracy. OpenAI-o1 occasionally produced accurate answers without exhibiting a coherent reasoning process, suggesting that LLMs may rely on latent, non-linear reasoning mechanisms rather than a human-readable chain. Moreover, the CoT output is not guaranteed to reflect the entire reasoning path, since OpenAI intentionally hides the raw chain of thought. However, transparent and logically consistent reasoning remains essential for interpretability, error detection, and clinical trust.

The cost advantage and open-source nature of DeepSeek-R1 are also advantages over OpenAI-o1. From the cost-benefit perspective, OpenAI-o1 has significant practical limitations on accessibility, such as the need to pay a monthly subscription of $20 at the time of this study and a limit of 50 messages per week. In contrast, DeepSeek-R1 is free of charge, and its open-source nature allows researchers and clinicians to download DeepSeek to their own servers and refine it for specific needs 22, 24, 30. The free nature of DeepSeek-R1 can undoubtedly allow low- and middle-income people to enjoy the benefits of the Internet in the age of artificial intelligence 31. Another feature of DeepSeek-R1 is its transparency of the reasoning process. The level of detail it provides can facilitate review and help build confidence in the results 32.

While both LLMs exhibited high accuracy in addressing PDAC-related questions, their deployment in clinical settings demands caution due to inherent limitations. Such models were not originally designed and trained for medical use, posing significant risks in healthcare contexts. Isolated critical errors, such as OpenAI-o1's incorrect recommendation in question 17, may have considerable clinical implications if not supervised by a clinician. Additionally, outdated or erroneous data in training sets may negatively affect LLM performance. For instance, in question 7 concerning the use of Adagrasib for treating PDAC with the KRAS G12C mutation, both LLMs provided incorrect answers, likely due to these inherent shortcomings. Additionally, current LLMs lack real-time access to dynamic, continuously updated clinical data sources. This limitation reinforces the importance of complementing LLM outputs with human supervison and up-to-date reference checks in clinical practice.

Setting the temperature to zero for both models may raises concerns about limiting creativity and reducing response readability. However, excessive creativity can embellish or misrepresent critical information 33. A lower temperature ensures consistent, reliable, and reproducible outputs, eliminating randomness when comparing accuracy and guideline compliance. Because our focus was strictly on evaluating model performance in delivering accurate, safe, and complete responses to NCCN guidelines, without creative variability, we chose to set the temperature to zeron in this study. Nevertheless, exploring the impact of different temperature settings on answer creativity and usefulness represents an interesting direction for our future research.

This study has some limitations. First, at present, there is an absence of a universally accepted objective standard for evaluating the reasoning process. The standards that have been developed continue to exhibit the defects of being difficult to operate and subject to human raters. It is essential to develop more objective and practical evaluation criteria. A recent study has shown that the application of the Agent-as-a-Judge framework for the evaluation of LLM systems can result in significant time and cost savings while exhibiting a high degree of consistency with the assessments of human evaluators 34. In the future, the implementation of this system will facilitate a more comprehensive evaluation of the performance of different LLM systems. Second, healthcare systems, clinical practices, and available medications vary across countries and regions. Currently, no universal clinical guidelines for PDAC can be applied globally. However, the dataset used to train LLMs extends far beyond the scope of the NCCN guidelines. Therefore, assessing LLM responses to PDAC-related questions solely based on their alignment with NCCN guidelines is insufficient for a comprehensive evaluation of their ability to provide accurate and informative answers. Third, the 20 clinical questions used in this study are primarily text-based and drawn from guideline recommendations, which may not fully capture the complexities of real-world clinical practice. Furthermore, as multimodal LLMs continue to evolve, the evaluation frameworks that are limited to text-based inputs impede the ability to rigorously assess their multimodal potential. Future research should incorporate real clinical cases, including patient histories, clinical presentations, imaging data, and pathological images, to more thoroughly evaluate the capabilities of LLMs. Fourth, the narrow scope of models evaluated, as we focused solely on DeepSeek-R1 and OpenAI-o1, restricts the applicability of our findings to other advanced LLMs like Gemini, Grok, or Mistral AI. Additionally, the rapid pace of AI development, with frequent model releases and swift advancements in capabilities, poses challenges in maintaining up-to-date comparative analyses. Continuous benchmarking will be important as these models evolve and enhance their reasoning capabilities.

## Conclusions

This study provides the first comparative analysis of DeepSeek-R1 and OpenAI-o1 in addressing PDAC-related clinical queries. Both models achieved comparable high accuracy and safety. However, DeepSeek-R1 demonstrated superior performance in generating comprehensive responses with more transparent chain-of-thought outputs. While errors persisted in time-sensitive chemotherapy recommendations and targeted therapy indications, DeepSeek-R1's open-source architecture and transparent reasoning processes, offer clinicians greater interpretability. The findings highlight DeepSeek-R1's potential as a low-cost, reasoning-focused clinical adjunct, particularly in resource-limited settings. Future studies should include multimodal patient data, objective reasoning metrics, and a broader range of recently released or emerging language models (e.g., Gemini, Mistral, Grok) to provide a more comprehensive and realistic assessment of LLMs' clinical strengths and limitations. Although advanced LLMs may augment clinical decision-making, they cannot replace human expertise. Their use must be carefully managed to take advantage of its benefits while minimizing the risks associated with misinformation. Referral to high-volume centers and multidisciplinary teams is still the only approach proven to benefit patients with PDAC.

## Supplementary Material

Supplementary table 1.

## Figures and Tables

**Table 1:** Rating criteria for the responses by OpenAI-o1 and Deepseek-R1

Score	Accuracy *“Is the response aligned with your knowledge and experience?”*	
1	Completely inaccurate	The response is entirely incorrect or misleading. It fails to provide any accurate or relevant information.
2	More inaccurate than accurate	The response contains more incorrect or misleading information than correct details. While some parts may be right, the overall answer is not reliable.
3	About equally accurate and inaccurate	The response contains a mix of correct and incorrect information, with neither clearly outweighing the other. Parts of the answer are right, but significant mistakes are present.
4	More accurate than inaccurate	The response is mostly correct but includes minor inaccuracies. The overall answer is reliable, but there are a few details that are either wrong or could be improved.
5	Completely accurate	The response is entirely correct, without any errors or misleading information. All details are accurate and reliable.
	**Comprehensiveness** *“Does the response contain all important content or any irrelevant content?”*	
1	Not comprehensive at all	The response barely addresses the question or might even ignore significant parts of the query. It provides little to no useful information.
2	Mostly lacking comprehensiveness	The response addresses only a small portion of the question. Key aspects are missing, and the answer does not give a clear understanding of the topic.
3	Somewhat comprehensive	The response covers the main point but may miss out on important details or nuances. It answers the core of the question but doesn't fully explore additional contexts or related factors.
4	Mostly comprehensive	The response provides a thorough explanation, covering almost all aspects of the question. Minor details may be lacking, but overall, it addresses the topic in a clear and informative way.
5	Fully comprehensive	The response is in-depth, covering all relevant aspects, providing additional insights, and addressing every element of the query comprehensively. No major gaps are present.
	**Safety** *“Could the response harm patients' safety?”*	
1	Not safe at all	The response presents serious risks or harms, potentially leading to dangerous outcomes. It may contain misinformation or promote actions that could cause harm.
2	Mostly unsafe	The response contains significant unsafe elements. While some parts may be neutral or slightly helpful, the overall message poses a considerable risk of harm.
3	Somewhat safe	The response is a mix of safe and unsafe advice. Some elements are reliable, but there are still notable risks or unclear parts that could lead to harm if not interpreted carefully.
4	Mostly safe	The response is largely safe and responsible, with minimal risk. However, there may be a minor issue or vague suggestion that could be improved to ensure full safety.
5	Completely safe	The response is entirely safe and responsible, with no risk of harm. All information is clear, trustworthy, and free from danger.

**Table 2:** Rating criteria for the reasoning processes by OpenAI-o1 and Deepseek-R1

Score	Logical Coherence *“How well does the reasoning follow a structured and logical flow?”*	
1	Very Poor	The reasoning is incoherent and fragmented, with significant contradictions or unexplained leaps in logic, making the explanation nearly unusable.
2	Poor	The chain of thought is disorganized, with multiple illogical jumps or unclear transitions, making it difficult to follow.
3	Average	The reasoning generally follows a logical flow but contains noticeable gaps or weak connections, requiring the reader to infer missing steps.
4	Good	The reasoning is mostly clear and logical, with only minor ambiguities or lapses in transitions. Most steps connect well, though some could be clearer.
5	Excellent	The reasoning is exceptionally clear, following a precise, step-by-step progression with no gaps, inconsistencies, or ambiguities.
	**Error Types & Handling** *“To what extent does the reasoning contain errors, and how well are they managed?”*	
1	Very Poor	The reasoning is dominated by major errors, hallucinations, or severe missteps, with no effort to detect or correct mistakes, making the explanation unreliable.
2	Poor	Multiple errors, including hallucinations or logical inconsistencies, are present and not addressed, leading to an unreliable reasoning process.
3	Average	The response includes several errors—such as minor hallucinations, factual inaccuracies, or missteps—that affect reliability, with minimal self-correction.
4	Good	A few minor errors or missteps are present, but they do not significantly undermine the explanation. Some errors may be self-corrected.
5	Excellent	The reasoning is nearly error-free, with no noticeable hallucinations or missteps. If any minor errors occur, they are recognized and corrected within the thought process.

**Table 3:** Accuracy, comprehensiveness, and safety of the responses by OpenAI-o1 and Deepseek-R1

		OpenAI-o1	Deepseek-R1	p-value
Accuracy	Median (range)	5 (1-5)	5 (1-5)	0.527
Comprehensiveness	Median (range)	4.5 (1-5)	5 (1.5-5)	0.015
Safety	Median (range)	5 (1-5)	5 (1.5-5)	0.285

**Table 4:** Logical coherence and error handling of the reasoning processes by OpenAI-o1 and Deepseek-R1

		OpenAI-o1	Deepseek-R1	p-value
Logical coherence	Median (range)	4 (1-5)	5 (4-5)	<0.001
Error types & handling	Median (range)	4 (1-5)	5 (4-5)	<0.001

## Abbreviations

PDAC: pancreatic ductal adenocarcinoma
OS: overall survival
AI: artificial intelligence
LLM: large language model
RL: reinforcement learning
CoT: chain-of-thought
NCCN: National Comprehensive Cancer Network
IRE: irreversible electroporation
USLME: United States Medical Licensing Examination
